# The Relation Between Autistic Traits, the Degree of Synaesthesia, and Local/Global Visual Perception

**DOI:** 10.1007/s10803-019-04222-7

**Published:** 2019-09-14

**Authors:** Floor Burghoorn, Mark Dingemanse, Rob van Lier, Tessa M. van Leeuwen

**Affiliations:** 1 Donders Institute for Brain, Cognition and Behaviour, Radboud University, Montessorilaan 3, 6525 HR Nijmegen, The Netherlands; 2 Max Planck Institute for Psycholinguistics, Nijmegen, The Netherlands; 3 Centre for Language Studies, Radboud University, Nijmegen, The Netherlands

**Keywords:** Autism, Synaesthesia, Synesthesia, Neurotypical, Visual perception, Local/global

## Abstract

Synaesthesia is highly prevalent in autism spectrum disorder. We assessed the relation between the degree of autistic traits (Autism Spectrum Quotient, AQ) and the degree of synaesthesia in a neurotypical population, and hypothesized both are related to a local bias in visual perception. A positive correlation between total AQ scores and the degree of synaesthesia was found, extending previous studies in clinical populations. Consistent with our hypothesis, AQ-attention to detail scores were related to increased performance on an Embedded Figures Task and reduced susceptibility to visual illusions. We found no relation between autistic traits and performance on a motion coherence task, and no relation between synaesthesia and local visual perception. Possibly, this relation is reserved for supra-threshold synaesthetes.

**Electronic supplementary material:**

The online version of this article (10.1007/s10803-019-04222-7) contains supplementary material, which is available to authorized users.

## Introduction

Until quite recently, autism spectrum disorder (ASD) was mostly known as a neurodevelopmental condition causing social dysfunctions (American Psychiatric Association 2013). Even though sensory atypicalities in ASD had already been acknowledged by Kanner (1943), the importance of sensory and perceptual alterations in ASD has been emphasized only recently (e.g. Simmons et al. 2009). This has led to the inclusion of sensory dysregulation, i.e. hypersensitivity or hyposensitivity to sensory input, as a diagnostic criterion for ASD (American Psychiatric Association 2013).

A recent finding related to sensory atypicalities in autism is a frequent co-occurrence of ASD with synaesthesia, another neurodevelopmental condition. Synaesthesia, which can be translated from Greek as ‘joined perception’, is characterized by altered perceptual experiences: perceiving an inducing stimulus elicits an unusual concurrent sensation in the same or a different modality, e.g. letters automatically evoke a colour. Whereas the general prevalence of synaesthesia only lies around ~ 4% (Simner et al. 2006), approximately 20% of people with ASD have synaesthesia (Baron-Cohen et al. 2013; Neufeld et al. 2013). No studies have yet assessed the ASD prevalence in the synaesthesia population, and hence the strength and full extent of the relationship are not yet known. However, the relatively high synaesthesia prevalence in the ASD population and increased interest in sensory atypicalities in ASD have inspired studies investigating possible commonalities between synaesthesia and autism.

One commonality is that synaesthesia and ASD are both considered to be dimensional rather than categorical constructs, although for both conditions this issue remains continuously under debate. Over the last decades, an increasing amount of evidence has been collected that suggests ASD to fall on a continuum, ranging from a low to high degree of autistic traits (Baron-Cohen et al. 2001; De Groot and Van Strien 2017; Hoekstra et al. 2008). The quantitative nature of ASD has been termed the broader autism phenotype (BAP). Similarly, several researchers have theorized synaesthesia to be of a dimensional nature, with quantitative differences existing between individuals (Cohen Kadosh and Henik 2007). In line with this theory, studies have found evidence for a continuum of the strength of pitch-size (Bien et al. 2012) and color-vowel (Cuskley et al. 2019) associations in the non-synaesthetic population, suggesting that individual differences exist in how sensitive people are to commonalities in the combinations of sensory information in their environment. Other researchers, however, argue that there is a clear qualitative distinction between those who are synaesthetes, and those who are not (Ward 2019).

Since altered perception lies at the heart of synaesthesia, it has been suggested that synaesthesia and ASD share perceptual and sensory characteristics (e.g. Ward et al. 2017). A better understanding of similarities in perceptual functioning in ASD and synaesthesia may lead to novel insights into perceptual/sensory dysregulation in individuals with ASD. This may also have practical implications by aiding individuals with ASD (and their clinicians) to better understand and cope with their perceptual/sensory symptoms.

Several studies have directly compared sensory perception in synaesthesia and ASD. One aspect of sensory perception is sensory sensitivity, which is frequently atypical in individuals with ASD, e.g. characterized by hyper- and/or hyposensitivity to sounds or touch (for a review, see Schauder and Bennetto 2016). More recently, similar patterns of increased and decreased sensory sensitivity (measured with the Glasgow Sensory Questionnaire) were found in both ASD and synaesthesia (Ward et al. 2017, 2018; Van Leeuwen et al. 2019), revealing particularly strong hyper- and hyposensitivity to auditory stimulation in both conditions.

Another aspect of perception that is of particular interest for the synaesthesia-ASD co-occurrence revolves around local/global visual processing. Navon (1977) was the first to propose that whereas the majority of individuals have an inclination towards global processing (i.e. ‘global precedence’, extracting the general gist of an image), some individuals are biased towards local processing. These individuals might thus be prone to ‘seeing the trees before the forest’. Local/global visual processing is a widely studied topic in ASD research, as many studies point to a bias towards local processing in individuals with ASD (for a review, see Happé and Frith 2006). In 1994, Frith and Happé proposed the ‘weak central coherence theory’, which, in its most recent form, states that individuals with ASD have a ‘detail-focussed cognitive style’ (a local bias).

In line with the findings for autism, synaesthetes have scored higher than controls on the Attention-to-detail subscale of the Autism Spectrum Quotient (AQ) (Mealor et al. 2016; Van Leeuwen et al. 2019; Ward et al. 2017, 2018), a self-report questionnaire on autistic traits. This finding suggests a shared bias in local visual perception between ASD and synaesthesia. Ward et al. (2018) also showed that synaesthetes outperformed controls on two tests requiring attention to detail, extending the perceptual commonalities between ASD and synaesthesia from the phenomenal (self-report) level to functioning at cognitive tasks.

The goal of the present study is to shed more light on the cognitive functioning of individuals with ASD and/or synaesthesia. Building on prior work, we investigate the relation between the degree of synaesthesia, autistic traits, and performance on local/global visual perception tasks. Before introducing the present research in more detail, we discuss studies that have investigated local/global visual processing separately in either ASD or synaesthesia. In addition to elucidating perception in both conditions, these studies also point to potential underlying neural mechanisms that could drive a shared bias in local/global visual perception.

### Evidence for a Local Bias from the ASD and Synaesthesia Research Fields

Various different tasks and paradigms have been used to study local/global visual perception in ASD. First, individuals with ASD tend to perform superior to controls on the Embedded Figures Task (EFT), a task that requires participants to detect a target shape embedded in a complex context (e.g. Pellicano et al. 2006; Brosnan et al. 2012). Focusing on individual elements facilitates performance on this task.

Second, a local bias is related to reduced susceptibility to visual illusions. To illustrate, the Ebbinghaus illusion consists of one ‘core’ circle that is surrounded by other (smaller or larger) contextual circles. Integrating the contextual circles with the core stimulus influences the perceived size of the latter. Focusing on the core circle (local bias) predicts a reduced susceptibility to the illusion, which was indeed confirmed for ASD (Happé 1996, Bölte et al. (2007). Chouinard et al. (2013) found AQ scores in neurotypicals to be negatively related to susceptibility to the Müller-Lyer illusion. Not all recent studies were able to replicate these reports of reduced susceptibility in ASD, however (Chouinard et al. 2016, Manning et al. 2017), calling for more studies to be performed in this area.

Third, individuals with ASD showed impaired performance compared to controls on a motion coherence task (MCT), which requires identification of the global motion direction of a group of moving dots. Individuals with ASD were found to have an, on average, 10% higher motion coherence threshold than controls in this task, indicating global processing deficits (for a review, see Dakin and Frith 2005). Jackson et al. (2013) found that this effect was strongest when the ability to track single, local dots is limited and one is therefore forced to assess global movement. Finally, when individuals with ASD are presented with hierarchical stimuli (Navon 1977) they tend to attend to the local rather than the global level of these stimuli (Koldewyn et al. 2013; Muth et al. 2014).

A meta-analysis by Cribb et al. (2016) showed that the results pointing towards a local bias also hold for individuals who possess a high degree of autistic traits (i.e. neurotypicals). This supports the idea of autism as a dimensional (as opposed to categorical) construct, with autistic traits distributed across a spectrum.

However, for both clinical and subclinical samples, results regarding a local bias in ASD are not fully conclusive, with several studies failing to support it (e.g. Chouinard et al. 2016; Manning et al. 2017 for the visual illusions) and a large heterogeneity appearing in meta-analyses (Cribb et al. 2016; Muth et al. 2014; Van der Hallen et al. 2015). These discrepancies call for more studies on the relation between ASD and local bias.

In synaesthesia only a few studies have investigated local/global perception, but these do suggest that synaesthetes exhibit a local bias similar to individuals with ASD. First, Ward et al. (2018) found synaesthetes to outperform controls on the EFT. Synaesthetes were also more accurate than controls on a Change Blindness Test, which requires detecting small changes in a visual environment. Second, Janik McErlean et al. (2016) found that although synaesthetes outperformed non-synaesthetes in facial identity tasks that required featural (local) discriminations of facial features, they did not on tasks that require configural (global) face processing. Finally, Banissy et al. (2013) found grapheme-colour synaesthetes to have increased motion coherence thresholds in the MCT, suggesting a possible global processing deficit. So far, however, only these three studies have addressed local/global processing in synaesthesia; no studies have addressed the susceptibility to visual illusions in synaesthetes. More research is needed to replicate these findings, and to extend their results to other local/global processing tasks.

In addition to commonalities in performance on local/global perceptual tasks, studies into ASD and synaesthesia have found neural similarities related to visual perception, such as enhanced sensitivity of the parvocellular visual pathway (sensitive to fine detail and high contrast) (see Brown and Crewther 2017; Sutherland and Crewther 2010; Jackson et al. 2013 for ASD, and Barnett et al. 2008; Van Leeuwen et al. 2013 for synaesthesia), as well as increased local and decreased global cortical connectivity (see Just et al. 2012 for ASD, and Hänggi et al. 2011, for synaesthesia). These studies provide suggestions for the neural mechanisms that might underlie a shared local bias. However, it should be acknowledged that a direct causal relation between the shared neural mechanisms and performance on perceptual task has not been established so far. Hence, it is possible that the shared sensory and local/global perceptual characteristics are caused by different neural mechanisms in synaesthesia and ASD, respectively.

### Purpose of the Current Research

The present research has three goals. First, we examine the relation between the degree of autistic traits and synaesthesia in neurotypicals, attempting to extend on prior studies that showed synaesthesia to be more common in individuals with diagnosed ASD. Second, we seek to reduce the inconsistency of evidence regarding the weak central coherence theory by studying the relationship between local/global processing and the degree of autistic traits. Finally, the relationship between synaesthesia and local/global processing abilities is examined in more detail.

Two features of the current study distinguish it from previous research in this field. First, the study is performed in a neurotypical population (i.e. in a population of individuals not classified as having synaesthesia or ASD), using continuous measures of the degree of synaesthesia and of autistic traits and thereby treating ASD and synaesthesia as dimensional constructs (Baron-Cohen et al. 2001; Cohen Kadosh and Henik 2007; Cuskley et al. 2019; De Groot and Van Strien 2017). Second, the current study is the first to specifically assess local/global perception in relation to both autistic traits and synaesthesia in the same study population.

### Study Approach and Hypotheses

The degree of autistic traits was measured by the Autism Spectrum Quotient (AQ; Baron-Cohen et al. 2001). The degree of grapheme-colour synaesthesia was measured by an extensive grapheme-colour synaesthesia consistency test (Eagleman et al. 2007). Given our focus on local/global visual perception, the Attention to detail-subscale of the AQ (AQ-detail) was of particular interest. We hypothesized that synaesthesia scores and AQ-detail scores would correlate positively, given previous findings (Mealor et al. 2016; Van Leeuwen et al. 2019; Ward et al. 2017, 2018). In addition, we expected synaesthesia scores to be positively correlated with AQ-total scores. This latter correlation was investigated to gain insight into the relation between the overall AQ-construct (formed of several subscales) and synaesthesia scores. A second reason for including AQ-total in our analyses is that although previous research has found the relation between synaesthesia and ASD to be strongest for the Attention to detail-subscale, synaesthetes have also been found to obtain elevated scores on the remaining four subscales (Van Leeuwen et al. 2019; Ward et al. 2017), especially when these were added together into an ‘AQ-other’ score (Ward et al. 2018).

Three experiments were devised to measure local/global visual perception. Experiment 1 was a motion coherence task, requiring participants to detect the global motion direction of a group of moving dots (MCT; Newsome and Paré 1988). This task can be generally performed using one of two strategies. With a ‘global strategy’, one focuses on the global movement of all dots together. With a ‘local strategy’, however, one picks a single dot and tracks this to identify its direction. For the present study, we created two task conditions, one with a limited (60 ms) and one with an unlimited (600 ms) dot lifetime. In the limited dot lifetime condition, participants are forced to use the global strategy, as the dot lifetime is too short to track single dots. Because of the hypothesized local bias, we expect a higher degree of autistic traits and synaesthesia to be related to impaired performance in this condition. In contrast, in the unlimited dot lifetime condition, participants can use a local strategy. In line with results from Jackson et al. (2013), we expect higher AQ/synaesthesia scores to be related to increased performance in this condition. That is, we hypothesize that because of their local bias, participants with higher AQ/synaesthesia scores are better at tracking single dots than participants with lower AQ/synaesthesia scores. It should be noted that a local strategy does not guarantee accuracy; that is, because only a subset of the dots are moving in the same direction, there is a chance of selecting and tracking the ‘wrong’ dot. However, assuming that people with both low and high AQ/synaesthesia scores use the local strategy in the unlimited dot lifetime condition, this chance of picking the wrong dot is the same for everyone.

In the second experiment, an Embedded Figures Task (EFT; Witkin et al. 1971) was used to assess local visual perception. A small target shape needed to be identified within a complex background figure, requiring focus on local elements while ignoring the background context. It was expected that both an increased degree of autistic traits (AQ-detail and AQ-total) and an increased degree of synaesthesia would be related to superior performance (decreased reaction times and/or a lower error percentage) on this task.

In Experiment 3, we assessed the susceptibility to visual illusions using a method-of-adjustment task devised by Manning et al. (2017). Participants adjusted the size of a stimulus (either the Ebbinghaus or Müller-Lyer illusion, Fig. 3) to make it match a reference stimulus. This method is sensitive to the extent to which the illusion is being perceived and therefore gives a graded indication of the susceptibility to the illusion, contrary to a same/different judgment task (Manning et al. 2017). In addition to the main condition containing illusory stimuli, participants completed a control condition in which they had to adjust context-free stimuli (e.g. simple circles). It is assumed that only in the illusory context condition, a local bias would aid performance, as this facilitates separating the illusory context from the ‘core’ of the stimulus. Using both types of conditions allowed us to control for potentially confounding factors of the relation between the degree of synaesthesia/autistic traits and visual perception, such as general increased/decreased perceptual functioning, or an increased/decreased motivation for accuracy. We predicted that only in the context condition, a higher degree of synaesthesia and autistic traits (AQ-total and AQ-detail) would be related to a smaller discrepancy between the to-be-adjusted stimulus and the reference stimulus, indicating a decreased susceptibility to visual illusions. In the context-free trials, we expected no relation between the degree of synaesthesia/autistic traits and performance.

## Methods

### Participants

At the time of study design, research on local/global visual perception in synaesthesia was minimally available, and a large heterogeneity existed in reported effect sizes in ASD (Cribb et al. 2016; Muth et  al. 2014; Van der Hallen et al. 2015). Therefore, it was difficult to determine an expected effect size for a priori sample size calculations. Using a medium effect size (*r* = .40 for correlations and *f* = .25 for repeated measures ANOVAs; Cohen 1988) in these calculations (G*Power; Faul et al. 2007), we included 39 participants to aim for a power of 0.80. These participants (of which *N* = 26 university students) took part in the study after providing informed written consent. Three participants were excluded due to neurological/psychiatric disorders, leaving 36 participants for analyses (10 males, mean age 25.47, *SD *= 9.07). Participants were recruited via an online study participation website and flyers around the university campus. Participation was compensated with 12.50 euros or 1.5 course credits. Informed consent was obtained from all individual participants included in the study. The study was approved by the Ethics Committee of the Faculty of Social Sciences (ECSW) at the Radboud University Nijmegen.

#### Degree of Autistic Traits

The degree of autistic traits was measured by the Dutch version of the Autism Spectrum Quotient (AQ-NL; Hoekstra et al. 2008), a self-report questionnaire (Baron-Cohen et al. 2001) providing a continuous measure of autistic traits in adults with normal intelligence. It consists of 50 statements concerning personal habits and preferences that can be agreed or disagreed with on a 4-point Likert scale (definitely agree, slightly agree, slightly disagree, and definitely disagree): e.g. item 13 states ‘I would rather go to a library than to a party’. Subscores are available for five subscales: Social skills, Communication, Imagination, Attention to detail, and Attention switching. The total AQ score is the sum of the subscores; higher scores indicate a higher degree of autistic traits. For the AQ-NL, total AQ scores lie between 50 and 200; each subscore has a potential minimum of 10 and a maximum of 50. Since we only predicted that AQ-total and AQ-detail would relate to a higher degree of synaesthesia and a bias towards local visual perception we focussed on these two scores.

The AQ-NL has been validated in a Dutch population by Hoekstra et al. (2008), evaluating its criterion validity, internal consistency reliability (α = .81) and test–retest reliability (α = .78) to be satisfactory. Since none of the 36 participants had been diagnosed with ASD, and the AQ cannot be used to diagnose people but only indicates a person’s position on the autism spectrum scale, no participants were excluded based on their scores. Participants completed the AQ in LimeSurvey (https://www.limesurvey.org/), which was run on the laboratory computer.

#### Degree of Synaesthesia

The extent to which participants experienced grapheme-colour synaesthesia was assessed with an online grapheme-colour synaesthesia test similar to the Synesthesia Battery (Eagleman et al. 2007). The test was developed for a ‘Groot Nationaal Onderzoek’ (Large National Survey), a crowdsourcing initiative in the Netherlands (http://gno.mpi.nl/test/synesthesietest; Van Leeuwen and Dingemanse 2016). Participants were serially presented with the numbers 0–9 and letters A–Z in random order, and were asked to indicate on a colour spectrum with which colour they associated this number or letter. All items were presented three times. Differences in RGB value between the three instances of each grapheme were used to compute a difference score that indicates the consistency of the associated colour experience. Difference score values below 1.43 are considered to signal consistent synaesthetic color experiences, a cut-off that was established in a test of specificity and sensitivity (Rothen et al. 2013) of the widely used Synaesthesia Battery (Eagleman et al. 2007), which is generally accepted as a classification tool for synaesthesia. Lower scores thus indicate a higher degree of synaesthesia.

Since this study investigated visual perception in a population of neurotypical individuals, we excluded participants classified as synaesthetes from further analyses. This prevented the potential association between synaesthesia scores, AQ scores and visual perception to be potentially inflated by a few influential scores at the higher end of the continuum (caused by synaesthetes), while the majority of the scores might be at a lower level. The cut-off for synaesthesia was at 1.43, in line with Rothen et al. (2013). To ensure that achievement of this score was due to the actual conscious experience of synaesthesia (rather than, for instance, a mere high memory performance), all participants completed a short post-test questionnaire on which they indicated whether or not they experienced any form of synaesthesia in daily life (see Supplementary Material). Participants who scored below 1.43 on the test *and* indicated experiencing synaesthesia in daily life were subsequently excluded from all analyses (*N *= 3). One other participant obtained a score within the synaesthetic range (1.27), but because this individual did not report any synaesthetic experiences they were retained in the sample.

Additionally, 4 of the 33 remaining participants failed to complete the synaesthesia test correctly (i.e. they did not pick any associating colours, or chose black or white for all items) and no synaesthesia score could be determined. These 4 participants were excluded from analyses concerned with the degree of synaesthesia. Therefore, all analyses related to the degree of synaesthesia were performed with *N *= 29. The 4 participants who failed to complete the synaesthesia test did complete the AQ, which resulted in a sample of *N *= 33 participants for the analyses concerning autistic traits and visual perception. For these reasons, it was decided that for each experiment, two separate analyses would be run: one with the AQ scores (in which *N *= 33), and one with the synaesthesia scores (*N *= 29).

### General Procedure

The experiment consisted of one 75-min laboratory session, in which all tests and questionnaires were administered. After providing informed consent, participants received instructions on the general procedure. The complete experiment was presented on a 24″ BenQ screen with display resolution set to 1920 × 1080, controlled by a Dell laboratory computer running Windows 7. Distance to the computer screen was 50 cm. Participants first performed the motion coherence task, followed by the Embedded Figures Task and the visual illusions task. After these three tasks were performed, the synaesthesia test and AQ questionnaire were completed immediately after each other. At the end of the experiment, participants were debriefed on the research’s purpose and hypothesis and thanked for their participation.

### Experiment 1: Motion Coherence Task (MCT)

#### Apparatus

Participants performed an online motion coherence task on the laboratory computer. This test was developed in HTML 5 (HTML/CSS/JavaScript), accessible via a URL specifying the task parameters, and run in Google Chrome.

#### Stimuli

**Fig. 1:**
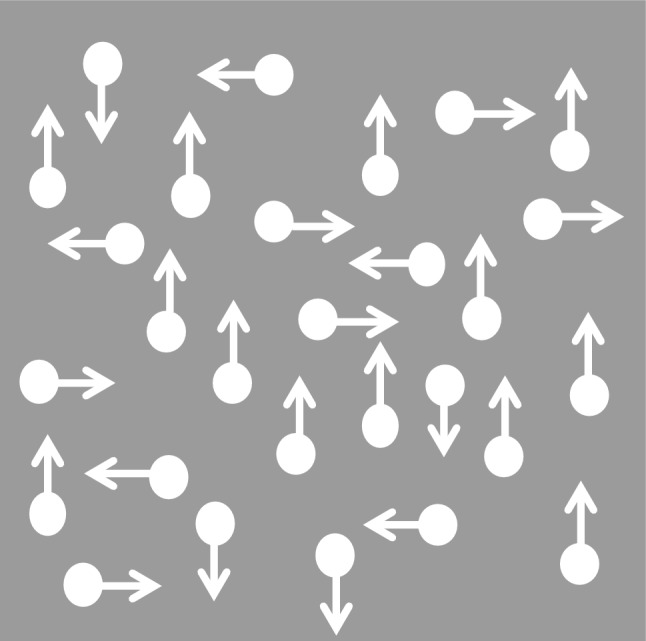
Schematic overview of the MCT. A subset of the presented dots moved in a coherent direction; this direction had to be identified by the participant

The visual display consisted of 200 white dots (diameter 0.15°) that moved with a speed of 6°/s across a 11.7 by 11.7 cm grey square background (see Fig. 1). A subset of the dots moved in a coherent direction, either rightwards (0°), downwards (90°), leftwards (180°) or upwards (270°). Two different dot lifetimes conditions were created. In the limited dot lifetime condition individual dots could be tracked to a limited extent (60 ms) (see Simmons et al. 2009, Table 1, for an overview of MCT parameters in ASD studies). In the unlimited dot lifetime condition, individual dots could be tracked during the full duration of the trial (600 ms).

**Table 1:** Descriptive statistics

Measure	M	SD	Range
Synaesthesia/AQ			
Synaesthesia	2.26	0.44	1.27–3.15
AQ-total	107.39	11.67	89–136
AQ-detail	25.15	3.83	18–34
MCT^a^			
60 ms	0.28	0.18	0.07–0.77
600 ms	0.08	0.03	0.03–0.17
EFT^b^			
% errors (total)	12.54	6.74	0–26.56
DL 1	3.22	4.97	0–18.75
DL 2	3.41	5.87	0–25.00
DL 3	14.02	12.00	0–43.75
DL 4	29.73	15.15	0–56.25
RT (s) (total)	3.69	2.99	0.80–18.16
DL 1	1.88	0.63	0.80–18.16
DL 2	2.50	1.18	0.93–6.14
DL 3	4.04	2.58	1.08–12.21
DL 4	6.30	3.92	0.98–18.16
Visual illusions^c^			
Ebb-NC	1.72	0.52	0.70–2.89
Ebb-C	5.38	1.92	2.20–10.45
ML-NC	4.94	1.18	2.10–8.25
ML-C	21.04	10.78	8.15–48.46

^a^Motion coherence task: 60 ms = limited dot lifetime condition, 600 ms = unlimited dot lifetime condition

^b^Embedded Figures Task: DL = difficulty level; RT = response times (s)

^c^Visual illusions task: Ebb-NC = Ebbinghaus illusion-no context; Ebb-C = Ebbinghaus illusion-context; ML-NC = Müller-Lyer illusion-no context; ML-C = Müller-Lyer illusion-context

#### Procedure

At the start of each run the motion coherence level was set to 0.5 (i.e. 50% of dots moving coherently). Participants used the arrow keys on the keyboard to indicate the motion direction during or directly after each trial, after which the next trial began immediately. After a correct trial the coherence level for the next trial was reduced logarithmically by dividing the current coherence level by 10^0.1^ (power bel function), while after an incorrect answer the coherence level was multiplied by 10^0.1^, making the task easier. The minimum motion coherence value was close to 0, the maximum was 1.0. For each dot lifetime condition, three runs of 60 staircase trials each were completed. Each participant thus completed both conditions (2 × 180 trials). The order of dot lifetime conditions was counterbalanced across participants.

#### Data-Analysis

For both dot lifetime conditions (60 ms and 600 ms) separately, the motion coherence thresholds at the end of each run (trial 60, 120 and 180) were averaged and taken as an indication of performance level. After 60 trials, the staircase procedure has typically led to stable motion coherence thresholds.

Two repeated measures ANCOVAs (one with the AQ and one with the synaesthesia scores) were used to analyse the main and interaction effects of dot lifetime (60/600 ms), AQ-total and AQ-detail, and synaesthesia consistency scores on the motion coherence threshold (the dependent variable). For the interactions to be analysed, the AQ-total, AQ-detail and synaesthesia consistency scores were standardized and used as covariates in the analysis (as recommended by Ellis 2016).

### Experiment 2: Embedded Figures Task (EFT)

#### Apparatus

Participants completed an online Embedded Figures Task, of which the stimuli and procedure were developed at the University of Leuven (de-Wit et al. 2017). The experiment was programmed in HTML 5 (HTML/CSS/JavaScript), accessible via a URL specifying the task parameters, and run on Google Chrome.

#### Stimuli

**Fig. 2:**
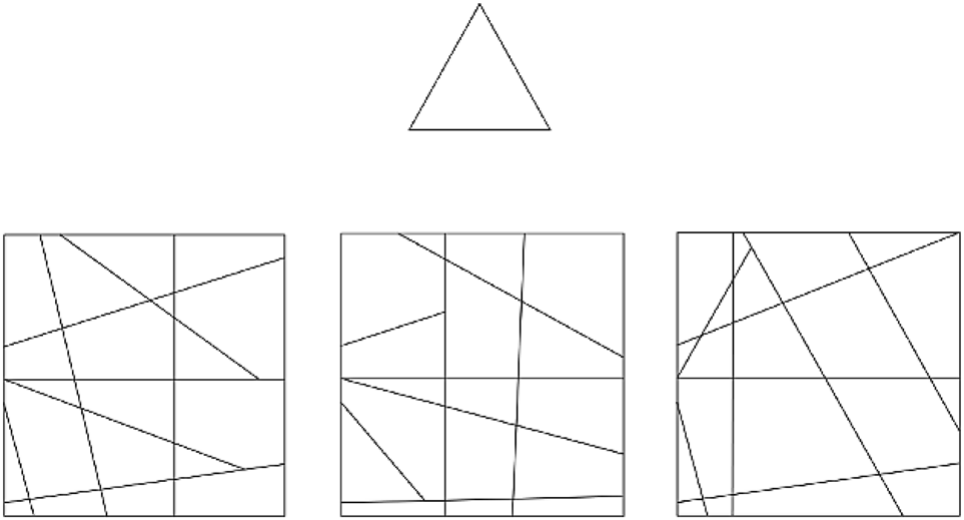
EFT visual display. The target shape is presented in the upper part of the display. This target shape is also hidden in one of the three complex figures at the bottom, in this case the right figure (at 66% continued lines, difficulty level 3) Adapted version of Fig. 2 of De-Wit et al. (2017) under a Creative Commons CC-BY 4.0 license (http://creativecommons.org/licenses/by/4.0/)

Each stimulus display consisted of a target shape at the top and three complex figures at the bottom of the display (Fig. 2). The general background was grey; the target shape and complex figures consisted of grey lines presented on white squares. The target shape was hidden in one of the three (left, middle or right) complex figures. The stimulus set consisted of 16 target shapes with their corresponding complex figures and each target shape was presented four times, at four different levels of difficulty. The difficulty manipulation was achieved by varying the number of target shape lines that continued into the background context of the complex figure. (0%, 34%, 64%, and 100% on average; for details see de-Wit et al. 2017). Thus, 16 trials of each difficulty level were presented in the experiment resulting in a total of 64 randomly presented experimental trials. Nine practice stimuli were used to familiarise the participants with the task.

#### Procedure

On each trial, participants clicked—as fast and as accurate as possible- on the complex figure in which they thought the target shape was hidden. Feedback was given on each trial: after a correct answer a green border appeared around the correct figure, and an arrow appeared allowing the participants to proceed to the next trial. After an incorrect answer a red border appeared around the selected figure, and participants had to try again until succeeding. To complete this session, all 64 trials had to be answered correctly.

#### Data Analysis

Error percentages and response times (RTs) were recorded for each participant. The overall error percentage was calculated as the percentage of the 64 trials that was answered incorrectly on the first presentation (i.e. number of errors/64 × 100%). RTs on incorrect trials were removed. RTs that were 1.5 interquartile ranges (IQRs) above the third quartile (75th percentile) for each difficulty level were denoted as outliers (Ellis 2014), and were removed from the analysis [4.42 trials (6.91%) on average]. The remaining response times were averaged per participant per difficulty level. The average RTs and error percentages were subsequently used as dependent variables in two doubly multivariate repeated measures ANCOVAs, with difficulty (1–4) as within-subjects factor and AQ-detail and AQ-total (in the first ANCOVA) and synaesthesia consistency scores (in the second ANCOVA) as covariates. Difficulty was incorporated in the analyses for exploratory purposes; to investigate whether the hypothesized main effects of AQ-detail, AQ-total and synaesthesia scores are dependent on the level of difficulty. The covariates were standardized to analyse the interaction between the covariates and the within-subjects factor (Ellis 2016).

### Experiment 3: Visual Illusions Task

#### Apparatus

The experiment was run in Matlab Psychtoolbox (Matlab R2015b 64-bit, Psychtoolbox 3.0.11, MathWorks). The method-of-adjustment task used was developed by Manning et al. (2017) for studying the susceptibility to visual illusions in autistic children. The task was adapted for the current research (e.g. number of trials, procedure).

#### Stimuli

**Fig. 3:**
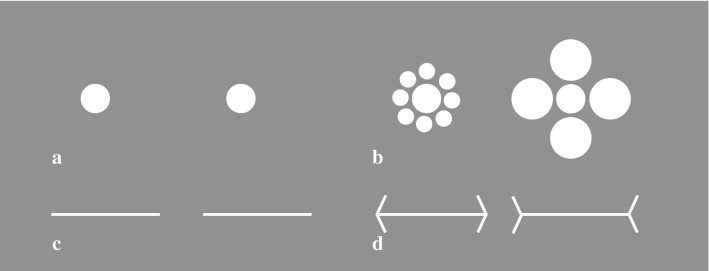
Visual illusion task visual display with stimulus examples. Both the Ebbinghaus and Müller-Lyer stimuli were presented without (**a**, **c**) or with (**b**, **d**) surrounding context. In the Ebbinghaus task (**a**, **b**), participants matched the size of the to-be-adjusted inner (**b**) or single (**a**) circles to the other circle. In the Müller-Lyer task, the to-be-adjusted inner (**d**) or single (**c**) lines had to be matched to the other line. The to-be-adjusted stimuli could occur on either side of the display. In this figure, all to-be-adjusted stimuli are identical in size to the reference stimulus. Adjusted from Figs. 1 and 2 from Manning et al. (2017) permitted under a Creative Commons CC-BY 4.0 license (http://creativecommons.org/licenses/by/4.0/)

The experiment consisted of two different tasks: an Ebbinghaus task (Fig. 3a, b) and a Müller-Lyer task (Fig. 3c, d). In both tasks, the visual display consisted of two side-by-side white stimuli (RGB level 250): either two Ebbinghaus stimuli or two Müller-Lyer stimuli, on a grey (RGB level 70) background. One of the two stimuli was the reference stimulus, the other was the to-be-adjusted stimulus. In the Ebbinghaus task, the central circle of the reference stimulus had a fixed diameter of 1.25°. This central circle was either surrounded by eight small context circles (diameter .42°) that had a distance of 1.25° from the centre stimulus, or by four large context circles (diameter 1.67°) that had a distance of 2.08° from the centre stimulus. The starting diameter of the central circle of the to-be-adjusted stimulus randomly varied between .68° and 1.82°. In the Müller-Lyer task, the reference stimulus consisted of a horizontal line (length 3°) with inward or outward context arrows at the end (at an angle of 45° compared to the horizontal line). The starting length of the to-be-adjusted stimulus randomly varied between 2.43° and 3.86°. The location of the reference and to-be-adjusted stimulus (left/right) was varied across trials. For both the Ebbinghaus and Müller-Lyer tasks, control (context-free) stimuli were created (Fig. 3a, c), which were identical to the experimental stimuli except that they had no contextual circles or arrows.

#### Procedure

At the start of each trial a small green rectangle (1000 ms) cued participants which of the two subsequently presented stimuli should be adjusted. Participants adjusted the size of the to-be-adjusted stimulus (using the up and down arrow keys) so that its size matched the reference stimulus and pressed the space bar to proceed to the next trial. There was no time limit and reaction times were not measured. Performance was defined as the physical discrepancy in pixels between the to-be-adjusted and reference stimulus, with a smaller discrepancy indicating higher performance and less susceptibility to the illusions.

Each participant performed four task sessions: two sessions of the Ebbinghaus task and two sessions of the Müller-Lyer task, in counterbalanced order. Each session consisted of one practice trial and 20 experimental trials. In the practice trials participants adjusted the size of a yellow star to make it match a reference star to familiarise themselves with the task. Of the 20 experimental trials per session 10 trials were context-free and 10 trials had context. The context- and context-free trials were blocked, and across the two sessions per task, it was counterbalanced whether participants started with 10 context-trials or 10 context-free trials. Within each 10 trials trial order was randomised. In the context-free trials two context-free circles or lines were presented; in the context-trials, two circles or lines with context were presented. In half of the trials per session, small circles (in the Ebbinghaus task) or inward arrows (in the Müller-Layer task) appeared on the left side of the screen and large circles (Ebbinghaus) or outward arrows (Müller-Lyer) appeared on the right side of the screen; in the other half, it was reversed.

#### Data Analysis

Preprocessing and data-analysis procedures were identical for the Ebbinghaus and Müller-Lyer task. First, performance on each trial was calculated by comparing the size of the to-be-adjusted stimulus with the size of the reference stimulus. Performance scores that were 1.5 interquartile ranges (IQRs) above the third quartile (75th percentile) were denoted as outliers (Ellis 2014), and were removed from the analysis (2.79 trials on average, i.e. 3.49%). Next, for each type of illusion separately, performance scores on all trials were averaged into one context-free score and one context score. This resulted in four measures per participant: Ebbinghaus-no context, Ebbinghaus-context, Müller-Lyer-no context, Müller-Lyer-context. These measures were used as dependent variables in a repeated measures ANCOVA, with context (present/absent) and type of illusion (Ebbinghaus/Müller-Lyer) as within-subjects factors and AQ-total and AQ-detail (in the first analysis) and synaesthesia consistency scores (in the second analysis) as covariates. For the interactions between AQ-total by context, AQ-detail by context and synaesthesia by context to be analysed, standardized values (*z*-scores) of AQ-total, AQ-detail and synaesthesia scores were used as covariates in the analysis (Ellis 2016).

## Results

Due to the separation of the analyses concerning the degree of autistic traits and synaesthesia (see methods section), the general task performance analyses (i.e. the effects of dot lifetime, type of illusion, difficulty) were performed twice. There were no differences between these results in terms of significant/non-significant effects; the only difference was that test statistics tended to be slightly smaller in the synaesthesia analyses due to the smaller sample size. To avoid unnecessary repetition, it was decided to only discuss the general task performance results as they were found in the AQ analyses. The descriptive statistics of all variables can be found in Table 1. All figures were created using the package ggplot2 (Wickham 2016) in R (R Core Team 2018) and Rstudio (R Studio Team 2016).

### The Degree of Autistic Traits and Synaesthesia

For descriptives, see Table 1. The AQ-total and AQ-detail scores were significantly correlated, *r*(31)= .50, *p *= .006, 95% CI [0.12, 0.77]. This is not surprising, since AQ-detail forms a subscore of the AQ-total. Using two related variables in the same analysis (when analysing the perception experiments) could lead to collinearity issues. This could reduce power, increase standard errors and potentially cause unstable estimates in the ANCOVAs (Baguley 2012; Dormann et al. 2013; Yoo et al. 2014). To examine this, we computed the variance inflation factor (VIF), a widely used measure of collinearity. The VIF was 1.28, amply below the critical value of 10 that is recommended by Dormann et al. (2013) and Yoo et al. (2014). In addition, we computed the tolerance, condition index (CI) and variance decomposition proportions (VD); none of these measure of collinearity exceeded the critical value described by Dormann et al. (2013). For this reason, and because analysing AQ-detail and AQ-total separately would increase the probability of a Type-I error, it was decided to retain both variables in the analyses.

Consistent with the participants’ self-reports, no AQ scores of 145 and above—the ASD cut-off score used by Hoekstra et al. (2008)—were obtained in our sample. Synaesthesia scores for all but one participant (see Methods) were within the non-synaesthetic range (*M *= 2.26), above the cut-off for synaesthesia (score of 1.43).

#### AQ-Synaesthesia Relation

**Fig. 4:**
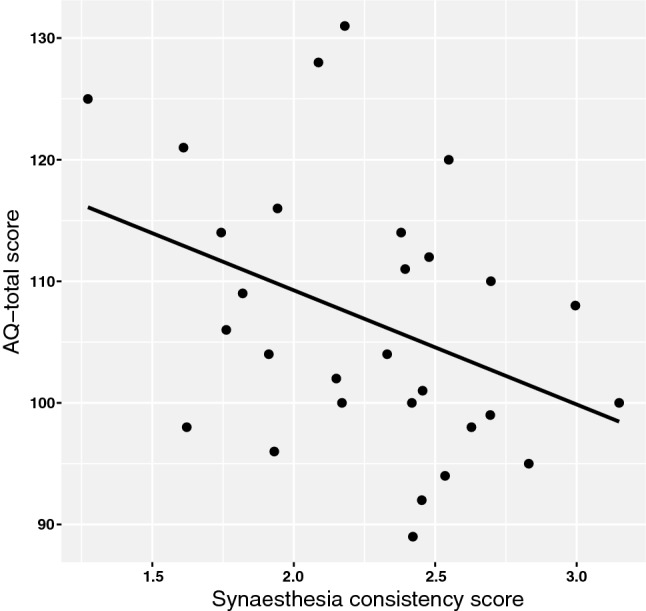
Correlation between the degree of autistic traits (the AQ-total score) and the degree of synaesthesia. Pearson’s *r *= − .38. Note: lower synaesthesia consistency scores indicate a higher degree of synaesthesia

The degree of synaesthesia and AQ-total correlated significantly, *r*(27)= − .38, *p *= .044, 95% CI [− 0.63, − 0.05], see Fig. 4 (note the correlation is negative because lower synaesthesia consistency scores indicate a higher degree of synaesthesia). This correlation indicates that a higher degree of synaesthesia is related to a higher degree of overall autistic traits. There was no significant correlation between synaesthesia and AQ-detail scores, *r*(27)= − .23 *p *= .23, 95% CI [− 0.57, 0.09].

### Experiment 1: Motion Coherence Task (MCT)

#### General Task Performance

The repeated measures ANCOVA showed a significant main effect of dot lifetime, *F*(1,30) = 43.57, *p *< .001, *η*^2^ = .59. The mean motion coherence threshold was higher in the limited (60 ms) dot lifetime condition than in the unlimited (600 ms) dot lifetime condition (Table 1). This reflects a general increase in performance with increasing dot lifetime.

#### Relation Between AQ Scores and Performance

There were no significant relations between AQ-total and AQ-detail and the overall motion coherence threshold (independent of dot lifetime, *F*(1,30) = 0.01, *p *= .934, and *F*(1,30) = 0.22, *p *= .640, respectively). Furthermore, no significant dot lifetime by AQ-total interaction, *F*(1,30) = 0.13, *p *= .726, nor dot lifetime by AQ-detail interaction, *F*(1,30) = 0.60, *p *= .446, was found. Thus, the relation between AQ-total/AQ-detail and the motion coherence threshold was not moderated by dot lifetime.

#### Relation Between Synaesthesia Scores and Performance

No significant relation between synaesthesia consistency scores and mean overall motion coherence thresholds was found, *F*(1,27) = 1.34, *p *= .256. Also, no significant synaesthesia by dot lifetime interaction was found, *F*(1,27) = 1.06, *p *= .313, indicating that the relation between synaesthesia consistency scores and mean motion coherence threshold did not differ for the two dot lifetime conditions.

### Experiment 2: Embedded Figures Task (EFT)

#### General Task Performance

Difficulty (levels 1–4) had a significant main effect on overall EFT performance, multivariate *F*(6,25) = 69.48, *p *< .001, *η*^2^ = .94. Univariate contrasts show that the differences in response times between level 1 and 2, *F*(6,30) = 79.21, *p *< .001, *η*^2^ = .73, 2 and 3, *F*(1,30) = 168.54, *p *< .001, *η*^2^ = .85, and 3 and 4, *F*(1,30) = 54.69, *p *< .001, *η*^2^ = .65, were all significant. Response times gradually increased as a function of difficulty level (Table 1). Error percentages did not differ between level 1 and 2, *F*(1,30) = 0.02, *p *= .881, whereas the differences between level 2 and 3, *F*(1,30) = 27.79, *p *< .001, *η*^2^ = .48, and 3 and 4, *F*(1,30) = 36.93, *p *< .001, *η*^2^ = .55, were significant. Similarly as for response times, higher difficulty levels led to higher error percentages (Table 1). The results are line with those reported in the original paper introducing this task (de-Wit et al. 2017).

Error percentages and response times did not correlate significantly, *r*(31)=− .16, *p *= .371, 95% CI [− 0.47, 0.08], so we have no indications for a speed-accuracy trade-off.

#### Relation Between AQ Scores and Performance

**Fig. 5:**
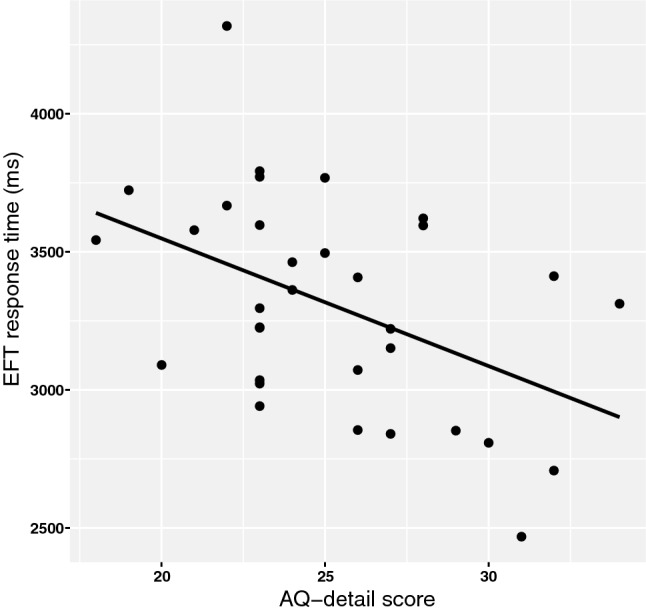
Correlation between the AQ-Attention to detail subscores and response times on the Embedded Figures Task. Pearson’s *r* = − .47, indicating faster response times with higher AQ-Detail

On a multivariate level, AQ-detail had a significant main effect on overall EFT performance, *F*(2,29) = 4.53, *p *= .019, *η*^2^ = .24; univariate tests showed this to be significant for response times, *F*(1,30) = 8.71, *p *= .006, *η*^2^ = .23, not for error percentages, *F*(1,30) = .001, *p *= .980, *η*^2^ < .001. A post hoc correlational analysis showed a negative relation between AQ-detail score and response times, *r*(31)= − .49, *p* = .004, 95% CI [− 0.71, − 0.13], indicating that higher AQ-detail scores were associated with faster performance (Fig. 5).

Next, on a multivariate level, AQ-total was not related to overall EFT performance, *F*(2,29) = 0.54, *p *= .589. Also, there were no significant multivariate interaction effects between AQ-detail and difficulty, *F*(6,25) = 1.17, *p *= .172, or between AQ-total and difficulty, *F*(2,29) = 0.75, *p *= .618. Therefore, the univariate tests of these analyses (which separated response times and error percentages) were not considered.

#### Relation Between Synaesthesia Scores and Performance

Synaesthesia consistency scores did not have a significant main effect on overall EFT performance, multivariate *F*(2,26) = 0.45, *p *= .641. Also, no significant interaction between synaesthesia consistency scores and difficulty was found, multivariate *F*(6,22) = 0.16, *p *= .958. Hence, univariate tests, separated on response time and percentages, were not considered.

### Experiment 3: Visual Illusions Task

#### General Task Performance

The repeated measures ANCOVA demonstrated a significant main effect of type of illusion, *F*(1,30) = 121.03, *p *< .001, *η*^*2*^=* .*80. The mean discrepancy between the to-be-adjusted and reference stimulus was lower in the Ebbinghaus task (*M *= 3.55, *SD *=1.02) than in the Müller-Lyer task (*M *= 12.99, *SD *= 5.28), reflecting overall higher performance in the Ebbinghaus than Müller-Lyer task.

Also, a significant main effect of context was found, *F*(1,30) = 91.11, *p *< .001, *η*^*2*^=* .*75, with the mean discrepancy between the two stimuli being higher in the contextual (*M *= 13.21, *SD *= 5.87) compared to the context-free (*M *= 3.33, *SD *= 0.73) trials, confirming the effectiveness of the contextual stimuli as visual illusions. Furthermore, a significant type of illusion by context interaction effect was found, *F*(1,33) = 60.70, *p *< .001, *η*^*2*^=* .*65. Post-hoc paired sampled t-tests revealed that the effect of context was significant in both the Ebbinghaus, *t*(32) = − 10,79, *p *= < .001, and Müller-Lyer, *t*(32) = − 8.30, *p *< .001 illusion. However, the effect of context was higher in the Müller-Lyer (mean difference = 16.09) than in the Ebbinghaus (mean difference = 3.66) task.

#### Relation Between AQ Scores and Performance

No main effect of AQ-total on the mean overall task performance (independent of the type of illusion and context) was found, *F*(1,30) = 0.79, *p *= .381. AQ-detail did have a marginal main effect on the overall task performance, *F*(1,30) = 3.87, *p *= .058, *η*^*2*^=* .*11 (medium effect size). Post-hoc correlational analysis on overall task performance revealed a trend indicating that a higher AQ-detail score was accompanied by a lower discrepancy between the to-be-adjusted and reference stimuli (i.e. a higher performance; *r*(31) = − .302, *p *= .088, 95% CI [− 0.55, − 0.01]).

**Fig. 6:**
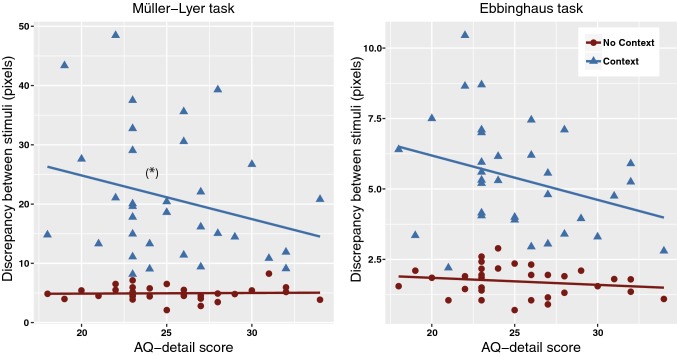
Significant interaction between context and AQ-detail on task performance in the visual illusions task, separated by the type of illusion. A lower discrepancy between the to-be-adjusted and reference stimulus indicates higher task performance. Results from the Müller-Lyer task (left panel) indicate that only in the context trails, a trend was found that relates a higher AQ-detail score to higher task performance. Results from the Ebbinghaus task (right panel) demonstrate no relation between AQ-detail and task performance in context or context-free trials

A significant context by AQ-detail interaction effect with medium effect size, *F*(1,30) = 4.21, *p *= .049, *η*^*2*^=* .*12, was found (Fig. 6). This is relevant because we hypothesized that AQ scores would only be related to performance in the context trials. Additionally, a marginal 3-way interaction between type of illusion, context and AQ-detail with medium effect size was present, *F*(1,30) = 3.66, *p *= .065, *η*^*2*^=* .*13. Examination of the regression coefficients revealed that in the Müller-Lyer task (Fig. 6a), there was a marginal relation between AQ-detail and performance only in the contextual trials (*b *= − 4.29, *SE* = 2.21, *t *= − 1.94, *p *= .062, *η*^*2*^=* .*11), not in the context-free trials (*b *= 0.27, *SE *= 0.24, *t *=1.13, *p *= .269). In the Ebbinghaus task (Fig. 6b), the relation between AQ-detail and the discrepancy between stimuli was not significant in the contextual (*b *= − 0.57, *SE* = 0.40, *t *= − 1.43, *p *= .163) nor context-free (*b *= − 0.07, *SE *= 0.11, *t *= − 0.64, *p *= .526) trials.

No interaction effects between AQ-total and context, *F*(1,30) = 1.69, *p *= .204, or other 2-way or 3-way interactions between the within-subjects and covariates were found.

#### Relation Between Synaesthesia Scores and Performance

**Fig. 7:**
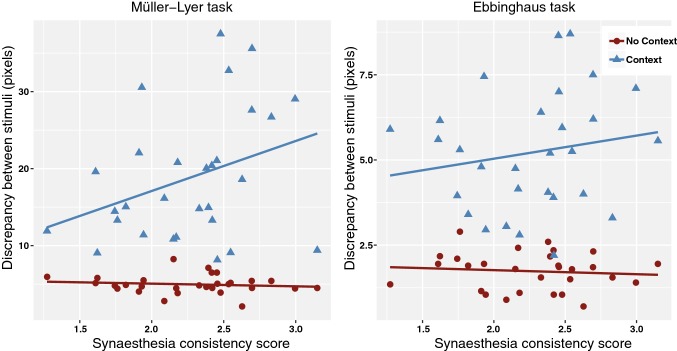
Interaction between context and synaesthesia consistency scores on task performance in the visual illusions task, separated by the type of illusion. Note that a lower discrepancy between to-be-adjusted and reference stimulus indicates higher task performance, and a lower synaesthesia consistency score indicates a higher degree of synaesthesia. In the Müller-Lyer task (left panel), a non-significant trend was found suggesting a positive relation between the degree of synaesthesia and task performance *only* in the context trials. No such trend was found in the Ebbinghaus task (right panel)

No significant main effect of synaesthesia consistency scores on mean overall task performance was found, although a trend was present, *F(*1,27) = 3.19, *p *= .085, *η*^*2*^=* .*11. There was a marginal interaction between context and synaesthesia scores with medium effect size, *F*(1,27) = 3.82, *p *= .061, *η*^*2*^= .12, while the 3-way interaction of context, synaesthesia score and type of illusion was not significant, *F*(1,27) = 3.13, *p *= .088, *η*^*2*^= .10. Figure 7 shows the pattern of performance in relation to the synaesthesia scores is similar to the relation between AQ-detail and task performance (Fig. 6). Because the effect size of the context by synaesthesia score interaction was as large as the effect size of the interaction effect for AQ-detail, and we hypothesized the relation between synaesthesia scores and performance would be similar to the relationship between AQ-scores and performance, we explored this similarity with the AQ results and examined the regression coefficients. It appeared that (analogous to the AQ results) a higher degree of synaesthesia was marginally related to a lower discrepancy between the to-be-adjusted and reference stimulus (i.e. higher task performance) only in the context trials of the Müller-Lyer illusion (in which *b* = 3.20, *SE *= 1.71, *t *= 1.88, *p *= .071, *η*^*2*^= .12). We note that this effect is not significant and cannot confirm a relation between synaesthesia scores and performance; all we can conclude is that the relation is in the same direction as for the AQ scores. No other 2- or 3-way interactions involving the synaesthesia scores were obtained.

## Discussion

We investigated the relation between the degree of autistic traits (as measured with the Autism Quotient) and the degree of grapheme-colour synaesthesia (as measured with a consistency test) in neurotypicals, and whether this relation is accompanied by a shared bias towards local (detail-focussed) visual perception. In line with our first hypothesis, a positive relation exists between the degree of autistic traits (AQ-total scores) and the degree of synaesthesia. In addition, and supporting our second hypothesis, a relation was found between the AQ-attention to detail subscores and a bias towards local visual perception, as indicated by performance on the Embedded Figures Task (EFT) and (to a lesser extent) the visual illusions task. Performance on the motion coherence task (MCT) was not related to AQ scores. Finally, no relation between the degree of synaesthesia and visual perception was found (with the exception of a non-significant trend in the visual illusions task resembling the results obtained for autism). This contradicts our hypothesis that synaesthesia would also be related to a tendency towards local visual perception. Therefore, two of our three main hypotheses were supported.

### The Degree of Synaesthesia and Autistic Traits

Our finding of a relation between autistic traits and the degree of synaesthesia in neurotypicals confirms research that previously established this link in clinical (i.e. supra-threshold) populations (Baron-Cohen et al. 2013; Neufeld et al. 2013). As far as we know this study is the first to report this relation in neurotypicals, supporting the idea of ASD and synaesthesia as dimensional constructs. The correlation between the degree of autistic traits and synaesthesia scores raises the question whether the ASD and synaesthesia continuums share certain characteristics. Based on the existing literature on visual perception in (clinical) ASD and synaesthesia, we hypothesized a local bias in visual perception might be shared. However, we found the synaesthesia scores to be related only to the AQ-total scores, not to the AQ-detail scores. The lack of a relation between the degree of synaesthesia and this subscale (assessing self-reported attention to detail) is in line with the results of our experiments on visual perception and its relation with synaesthesia scores, as explained below.

### The Degree of Autistic Traits and Visual Perception

Our results show a relation between the degree of autistic traits (AQ-detail scores, not AQ-total) and a local bias in visual perception on an embedded figures task and an illusion task. Our findings support prior studies that demonstrated a local bias in individuals with ASD, and are therefore in line with the detail-focussed cognitive style as proposed by the weak central coherence theory (Happé and Frith 2006). The results are also in line with the findings from Cribb et al. (2016), showing that this tendency towards detail-focused perception exists along the autistic (sub- and supra-clinical) spectrum.

The strongest evidence for the local bias was found in the EFT, as a higher degree of autistic traits was related to faster response times on this task. This is consistent with studies performed in clinical populations, which also found faster response times in individuals diagnosed with ASD compared to controls (Brosnan et al. 2012, Pellicano et al. 2006). Brosnan et al. (2012), however, also found fewer errors in individuals with ASD, which we could not confirm in our sample. We furthermore found a relation between AQ-detail scores and a reduced susceptibility to visual illusions, as indicated by a significant AQ-detail by context interaction. This points towards an increased ability to focus on relevant, local visual aspects and ignore contextual distractions. This is consistent with several previous studies (e.g. Bölte et al. 2007; Happé 1996). When breaking down this AQ-detail by context interaction for each visual illusion separately, we found a trend only in the Müller-Lyer (and not in the Ebbinghaus) illusion. This result is in accordance with Chouinard et al. (2013), who also reported a relation between AQ scores and susceptibility to the Müller-Lyer illusion, and acknowledge that there is no consensus on why this effect is only present in this particular type of illusion. One possible explanation could be that the Müller-Lyer illusion can be classified as a ‘within-object contextual illusion’, meaning that the contextual elements (the surrounding arrows) are physically attached to the local elements (the lines)—something that is not the case in the Ebbinghaus-illusion, which can be classified as a ‘between-object contextual illusion’ (Ben-Shalom and Ganel 2012). Therefore, it might be more difficult for most people to *not* integrate the context when perceiving the Müller-Lyer illusion, and having the ability to focus on the local elements (i.e. a local bias) could result in greater performance benefits on such a within-object illusion than in between-object contextual illusions. It should be noted that in Chouinard et al. (2013), the susceptibility to the Müller-Lyer illusion did not relate to AQ-Attention-to-detail (but only to total AQ scores).

The degree of autistic traits and performance on the motion coherence task (MCT) did not relate, and there was no moderation by dot lifetime. It is not clear whether enhanced local perception in ASD is indeed always accompanied by impaired global perception: although researchers traditionally supported this idea (Frith and Happé 1994), recent studies suggest that individuals with ASD merely have a reduced *preference* to process global information, not a reduced *ability* (Happé and Frith 2006; Koldewyn et al. 2013; Van der Hallen et al. 2015). This possibly explains why autistic traits did not relate to performance in the limited dot lifetime condition (60 ms) in which participants were forced to process global motion. In the unlimited dot lifetime (600 ms) condition, which allowed the tracking of single dots, we anticipated a positive relation between AQ-scores and performance. One potential reason for the lack of this relation might be that the assumption underlying our hypothesis was not correct. That is, we assumed that in the unlimited dot lifetime condition, participants with both low and high AQ-scores would use a ‘local strategy’ (i.e. tracking a single dot to identify its motion direction). However, it is also possible that participants with low AQ-scores refrained from using the local strategy altogether, and used the global strategy regardless of dot lifetime. Here, people with both low and high AQ-scores might have obtained high performance, but using different strategies. In addition, only people who used the local strategy (participants with high AQ-scores according to this line of reasoning) might sometimes have suffered from selecting and tracking the wrong dot, an error that is inherent to the local strategy. This may have prevented participants with high AQ-scores to outperform participants with lower AQ-scores on the unlimited dot lifetime condition.

Alternatively, a failure to find the hypothesized positive relation between AQ scores and performance in the unlimited dot lifetime condition could have been due to a ceiling effect. It is possible that the unlimited dot lifetime condition was too easy compared to the limited dot lifetime condition, resulting in all participants scoring significantly better and not allowing for a differentiation to be made based on AQ score. Similar results were obtained by Manning et al. (2015), who used dot lifetimes of 83 versus 1000 ms and found no moderating effect of dot lifetime. Jackson et al. (2013) used dot lifetimes of 80 and 300 ms and did find an AQ by dot lifetime interaction, supporting this explanation. Future studies are encouraged to systematically vary these dot lifetimes, to investigate in what range (between 50 and 600 ms) the dot lifetime forms a moderating effect on the relation between the degree of autistic traits and performance on the MCT.

Finally, one issue that should be discussed relates to our earlier discussion about the interdependence of AQ-detail and AQ-total, and the use of both variables in the same analyses. Although we have shown that the relation between these variables does not cause statistical collinearity issues, it should be acknowledged that AQ-detail and AQ-total are, by definition, not independent (as AQ-detail forms a subscore of AQ-total). Therefore, one might still wonder what the relation between the degree of autistic traits and visual perception looks like when this dependency between AQ-detail and AQ-total is excluded. In order to examine this, we reran our analyses in all three visual perception experiments, replacing AQ-total by an AQ-other score, a score composed of all AQ subscales except AQ-detail (similar to Ward et al. 2018). To be certain, we assessed the degree of collinearity between AQ-detail and AQ-other, which was found not to be problematic (e.g. VIF = 1.03). The results of the analyses were very similar to our previous results. For the MCT and EFT, we found no differences in results. For the visual illusions task, the only difference we found was that the multivariate AQ-detail by Context interaction became marginally significant [*F*(1,30) = 3.30, p = .079, *η*^*2*^=* .*10]. This shows that although the results are not completely similar, the degree of similarity to our previous results provides support for the relative stability of our initial estimates. This confirms that the interdependence between AQ-total and AQ-detail did not have a major influence on our results.

### The Degree of Synaesthesia and Visual Perception

In contrast to the findings for the degree of autistic traits, we did not find a relation between the degree of synaesthesia and local visual perception in neurotypicals. This result, combined with the fact that we found no correlation between the synaesthesia consistency scores and the AQ-detail scores, raises the possibility that the relation between the degree of synaesthesia and the AQ-total scores should be sought somewhere else than in a shared local bias. To investigate this possibility, we decided to explore the correlation between the synaesthesia scores and the remaining AQ-subscores separately (Social skills, Communication, Imagination and Attention switching), as well as when they were added together into an AQ-other subscale. However, no significant correlations were found, providing no support for this potential explanation.

We found a non-significant trend for the relation between the degree of synaesthesia and susceptibility to visual illusions that was very similar to what we found in the AQ analyses. This tentatively points towards an alternative explanation for our results that does not completely exclude a potential local bias in synaesthesia: a possible bias towards local visual perception could be stronger in supra-threshold synaesthetes. Support for this explanation stems from recent studies reporting a local bias in supra-threshold synaesthetes. Ward et al. (2018) and Van Leeuwen et al. (2019) found increased AQ-detail scores in supra-threshold synaesthetes, which we did not find in our non-synaesthete sample. Van Leeuwen et al. (2019) report decreased performance on the limited dot lifetime MCT in synaesthetes and both studies demonstrated a decreased error percentage on the EFT. Interestingly, however, they did not find decreased response times, which could potentially be explained by differences in testing procedure (online versus in the laboratory).

To see whether our own data could provide some further (speculative) support for the differences between sub- and supra-threshold synaesthetes, we explored the scores of the three synaesthete participants who were excluded from our neurotypical sample. Supporting previous research in synaesthetes (Ward et al. 2017) and extending on the correlation in neurotypicals, the AQ-total/synaesthesia correlation was maintained and even became more prominent when including these 3 participants, *r*(30)= − .48, *p *= .006, 95% CI [− 0.70, − 0.19]. This suggests that the relation between the degree of synaesthesia and autistic traits we found in neurotypicals can be extended to a sample including supra-threshold synaesthetes and encourages future studies to investigate the possible existence of a local bias along a synaesthesia continuum.

### Strengths and Limitations of the Present Study

Our study assessed visual perception using three different experiments, allowing for a balanced assessment of a potential local bias in visual perception. Furthermore, we systematically investigated certain task properties (e.g. dot lifetime, difficulty, type of illusion) that could potentially moderate the relation between the degree of autistic traits/synaesthesia and visual perception. This extends on previous reviews and meta-analyses that discussed the possible moderating influence of such task properties (see King et al. 2017 on visual illusions, and Simmons et al. 2009 on dot lifetime); possibly explaining inconsistencies in previous research as discussed in the introduction. Third, we used an extensive grapheme-colour synaesthesia test. By complementing these test scores with a self-report questionnaire on the possible experience of synaesthesia, we managed to exclude individuals with synaesthesia from our sample while still obtaining reliable measures of grapheme-colour consistency.

Our study also has some limitations, which are important for future studies to consider. First, we only assessed the degree of synaesthesia for the grapheme-colour type. The relation between the degree of synaesthesia and the degree of autistic traits might also extend to other types of synaesthesia: if so, then this relation could be ascribed to a more fundamental, underlying synaesthesia ‘trait’ (Rouw and Scholte 2016; Rouw et al. 2011). Future studies could investigate whether the relation between the degree of synaesthesia and local/global visual perception might be dependent on the specific synaesthesia type. For instance, using a questionnaire to assess local/global bias, Mealor et al. (2016) found evidence for a self-reported local bias in sequence-space synaesthesia, but not in grapheme-colour synaesthesia. Future studies could use visual perception tasks to confirm these findings, and could extend this to other types of synaesthesia. A second limitation of our study comes from the fact that, due to exclusion of several participants from our original sample (*N* = 39), we performed our analyses with a relatively small sample size (33 in the AQ analyses and 29 in the synaesthesia analyses). Although we did find significant effects in several of our analyses, the small sample size made it harder to reliably detect relatively subtle effects. Future studies could seek out to validate our results more firmly using a larger sample of participants.

Finally, a potential limitation concerns the use of a synaesthesia test as a continuous measure of the degree of synaesthesia in healthy controls. Since our participants did not actually have any synaesthesia, it should be noted that a high grapheme-colour consistency score might also reflect alternative underlying traits. For instance, it could indicate a high memory performance, a high attention to colours, or a high degree of conscientiousness. The latter possibility, however, would not account for the relation between the degree of synaesthesia and autistic traits that we found, as a recent meta-analysis (Lodi-Smith et al. 2018) found conscientiousness to be negatively correlation with ASD characteristics. This limitation concerns the widely discussed debate whether synaesthesia should be considered as a continuous traits or all-or-none phenomenon, as discussed in the introduction. Future research is encouraged to investigate the value of synaesthesia tests as a measure of the degree of synaesthesia in neurotypicals.

## Conclusions

Our study has provided insight into the perceptual experiences and abilities inherent to synaesthesia and ASD, as well as into their relationship. We found a positive relation between the degree of synaesthesia and autistic traits, one of the first demonstrations of this relation in a neurotypical sample. Furthermore, we found a relation between the degree of autistic traits and a bias towards local visual perception. We did not find evidence for a relation between the degree of synaesthesia and a bias towards local visual perception. One explanation for this last result might be that this local bias is expressed stronger in supra-threshold synaesthetes than in sub-threshold synaesthetes; this explanation is supported by other studies into synaesthetes and by a speculative exploration of our own data. Future studies are encouraged to study this alternative explanation, and/or to extend our research to other types of synaesthesia.

Gaining a better understanding of the relationship between ASD and synaesthesia can lead to important insights into perceptual alterations in ASD. This might also have practical implications; that is, as perceptual dysregulation has been acknowledged as an important clinical feature of ASD, an increased understanding hereof might aid individuals with ASD, as well as their clinicians.

## Electronic Supplementary Material

Below is the link to the electronic supplementary material.

Supplementary material 1 (PDF 111 kb)

## Data Availability

All data associated with this paper can be retrieved from: http://hdl.handle.net/11633/aacblxq5
