# Supplementary material for: The Relation Between Autistic Traits, the Degree of Synaesthesia, and Local/Global Visual Perception
Source: J Autism Dev Disord. 2019 Sep 14;50(1):12–29. doi: 10.1007/s10803-019-04222-7 (PMC6946735; doi:10.1007/s10803-019-04222-7)
Supplement: Supplementary file 1 — Supplementary material 1 (PDF 111 kb) [file 10803_2019_4222_MOESM1_ESM.pdf]

Supplementary Material

The Relation Between Autistic Traits, the Degree of Synaesthesia, and Local/Global Visual Perception

Floor Burghoorn[1], Mark Dingemanse[1,2,3], Rob van Lier[1], Tessa M. van Leeuwen[1,*]

1) Donders Institute for Brain, Cognition and Behaviour, Radboud University, Nijmegen, the Netherlands.
2) Max Planck Institute for Psycholinguistics, Nijmegen, the Netherlands.
3) Centre for Language Studies, Radboud University, Nijmegen, the Netherlands.

* Corresponding author: tesvlee@gmail.com

Post-test synaesthesia questionnaire

We report the post-test synaesthesia questionnaire that was used to verify that people who achieved a synaesthesia consistency score of below 1.35 also experienced synaesthetes in real life. For the study, a Dutch version of this questionnaire was used.

1. Which type or which types of synaesthesia do you experience?
   - Letters or numbers elicit certain colours
   - Days of the week or months of the year elicit certain colours
   - I see colours when I listen to music
   - I experience days of the week or months of the year in a certain spatial configuration (for example, a circle, oval or rectangle).
   - When other people are being touched, I feel this touch myself

2. Do you experience other forms of synaesthesia that were not mentioned in the previous question? Or do you have any comments about the type(s) of synaesthesia that you marked in the previous question?
[Open-ended question]

3. Do the ‘elicitors’ (e.g., the numbers, letters, months or touches) *always* elicit synaesthesia?
   - Yes
   - No
   - Not applicable

4. Is your synesthetic experience always the same? (For example: does Monday always elicit the colour red?)
   - Yes
   - No
   - Not applicable

5. As how strong would you rate your synaesthetic experience, on a scale from 1 to 10? You can skip this question if you do not experience synaesthesia.
[Open-ended question, only numbers allowed]
6. Do your synaesthetic experiences occupy a position in your environment (e.g., you feel them on your body, or you see them on words or objects in your environment), or do you experience them in your mind?

- They occupy a position the environment
- They are in my mind
- Not applicable

7. Have you experienced synaesthesia your whole life? If not, please indicate (approximately) since when you have experienced synaesthesia.

- Yes
- No
- Not applicable

8. Do close family members (parents/siblings) experience any form of synaesthesia?

- Yes
- No
- Not applicable
